# In-hospital mortality and cardiovascular treatment during hospitalization for heart failure among patients with schizophrenia: a nationwide cohort study

**DOI:** 10.1017/S2045796023000744

**Published:** 2023-10-18

**Authors:** Masahiro Nishi, Akira Shikuma, Tomotsugu Seki, Go Horiguchi, Satoaki Matoba

**Affiliations:** 1 Department of Cardiovascular Medicine, Graduate School of Medical Science, Kyoto Prefectural University of Medicine, Kyoto, Japan; 2 Department of Biostatistics, Kyoto Prefectural University of Medicine, Kyoto, Japan

**Keywords:** cardiovascular disease, heart failure, in-hospital mortality, mental health, outcome studies, quality of care, schizophrenia

## Abstract

**Aims:**

Schizophrenia is associated with cardiovascular disease (CVD) risk, and patients with schizophrenia are more likely to receive suboptimal care for CVD. However, there is limited knowledge regarding in-hospital prognosis and quality of care for patients with schizophrenia hospitalized for heart failure (HF). This study sought to elucidate the association between schizophrenia and in-hospital mortality, as well as cardiovascular treatment in patients hospitalized with HF.

**Methods:**

Using the nationwide cardiovascular registry data in Japan, a total of 704,193 patients hospitalized with HF from 2012 to 2019 were included and stratified by age: young age, > 18 to 45 years (*n* = 20,289); middle age, >45 to 65 years (*n* = 114,947); and old age, >65 to 85 years (*n* = 568,957). All and 30-day in-hospital mortality as well as prescription of cardiovascular medications were assessed. After multiple imputation for missing values, mixed-effect multivariable logistic regression analysis was performed using patient and hospital characteristics with hospital identifier as a variable with random effects.

**Results:**

Patients with schizophrenia were more likely to experience prolonged hospital stays, and incur higher hospitalization costs. In-hospital mortality for non-elderly patients with schizophrenia was significantly worse than for those without schizophrenia: the mortality rate was 7.6% vs 3.5% and the adjusted odds ratio (OR) was 1.96 (95% confidence interval (CI): 1.24–3.10, *P* = 0.0037) in young adult patients; 6.2% vs 4.0% and 1.49 (95% CI: 1.17–1.88, *P* < 0.001) in middle-aged patients. Thirty-day in-hospital mortality was significantly worse in middle-aged patients: the mortality rate was 4.7% vs 3.0% and an adjusted OR was 1.40 (95% CI: 1.07–1.83, *P* = 0.012). In-hospital mortality in elderly patients did not differ between those with and without schizophrenia. Prescriptions of beta-blockers and angiotensin-converting enzyme inhibitors or angiotensin II receptor blockers were significantly lower in patients with schizophrenia across all age groups.

**Conclusion:**

Schizophrenia was identified as a risk factor for in-hospital mortality and reduced prescription of cardioprotective medications in non-elderly patients hospitalized with HF. These findings highlight the necessity for differentiated care and management of HF in patients with severe mental illnesses.

## Introduction

Heart failure (HF) imposes enormous medical and economic burden on society, owing to its high morbidity and mortality rate (Storrow *et al.*, 2014; Virani *et al.*, 2021). The aetiology and exacerbating factors for HF involve a complex interplay of genetic or congenital diseases, ageing, lifestyle-related factors, atherosclerosis, structural degeneration and other acquired diseases. Effective management for those risk factors, while maintaining quality of care, is crucial for preventing the onset and improving the clinical prognosis of HF.

Severe mental illnesses, such as schizophrenia and depression, are associated with cardiovascular disease (CVD) (Correll *et al.*, 2017; Goldfarb *et al.*, 2022). Individuals with schizophrenia have a shorter lifespan than the general population, largely due to an elevated mortality risk of CVD (Hjorthoj *et al.*, 2017; Ringen *et al.*, 2014). Indeed, schizophrenia has been shown to increase major cardiac events following acute coronary syndrome (Attar *et al.*, 2019; Hauck *et al.*, 2020) and mortality in patients with HF (Jorgensen *et al.*, 2017; Polcwiartek *et al.*, 2021). Several causal factors have been proposed to substantiate the increased CVD risk in patients with schizophrenia: genetic predisposition, lifestyle factors including physical inactivity, unhealthy diet, smoking, substance use, obesity and metabolic or arrhythmogenic side effect of antipsychotics (Ringen *et al.*, 2014; Veeneman *et al.*, 2022). In addition, patients with schizophrenia are more likely to receive suboptimal care for CVD, characterized by low prescription rates of cardioprotective medications, reduced indication for invasive treatment and inadequate access to primary healthcare (Attar *et al.*, 2020; Goldfarb *et al.*, 2022; Kugathasan *et al.*, 2018).

Despite increasing evidence for the association between schizophrenia and CVD risk, there is limited knowledge regarding in-hospital prognosis and quality of care for patients with schizophrenia hospitalized due to HF. In this study, we sought to elucidate the association between schizophrenia and in-hospital mortality, as well as cardiovascular treatment in age-stratified patients hospitalized with HF, using nationwide registry data of CVD.

## Methods

### Study design

We used a nationwide cardiovascular registry data, the “Japanese Registry of All Cardiac and Vascular Diseases (JROAD) – Diagnostics Procedure Combination (DPC)” data, collected from hospitals specializing in cardiovascular care by the Japanese Circulation Society to investigate the clinical performance for patients with CVD in Japan (Nishi *et al.*, 2023; Yasuda *et al.*, 2018). The JROAD data, which included hospital characteristics, was linked to DPC data, medical claim data for acute-care hospitalization, comprised patient characteristics and clinical data including medications and procedures. Diagnoses and comorbidities were selected according to the International Classification of Disease and Related Health Problems, Tenth Revision (ICD-10) codes; I500, 501, 509 for HF, and F20–29 for schizophrenia. The data were collected from 2012 to 2019. We enrolled a total of 708,143 patients aged ≥18 to 85 years admitted with acute or chronic HF as a primary diagnosis for admission in 1,074 hospitals. We excluded patients who survived and were discharged within 1 day. When the missing rate of a variable was less than 1%, we excluded the patients with that missing data. Lastly 704,193 patients in 1,065 hospitals were included. The study was approved by the ethics committee of Kyoto Prefectural University of Medicine (approval number ERB-C-2194). This study conformed to the principles outlined in the Declaration of Helsinki.

### Statistics

All statistical analyses were conducted using R version 4.2.0. (R Core Team, 2022) All primary analyses were performed after imputation of missing values. Some variables, such as body mass index (BMI), systolic blood pressure, heart rate, New York Heart Association (NYHA) class, cardiovascular rehabilitation per year and hospital training status, contained missing values >5%. Data were assumed to be missing at random. Using all variables including outcomes, multiple imputation by means of chained equations was performed to generate 20 replications (Sterne *et al.*, 2009). Specifically, we used mice function from mice package with the arguments of *m* (number of multiple imputations) = 20 and maxit (number of iterations) = 20. All the replicated datasets were bound by complete function.

Subsequently, the data were stratified by each age group. Baseline characteristics including length of stay and hospitalization were described for each age group. We implemented mixed-effect logistic regression model by glmer function from lme4 package to analyse all and 30-day in-hospital mortality, and prescription of cardioprotective medications in patients hospitalized with HF by multivariables including patient characteristics (age, sex, BMI, smoking history, Charlson score, NYHA class, comorbidity of schizophrenia, heart rate and systolic blood pressure) and hospital characteristics (annual hospitalizations with HF per hospital, and hospital training status). Hospital identifier (ID) was incorporated as a variable with random effects towards intercepts. The estimates were combined by pool function. Two-sided *P* value <0.05 was considered statistically significant.

## Results

### Participants

**Figure 1.**
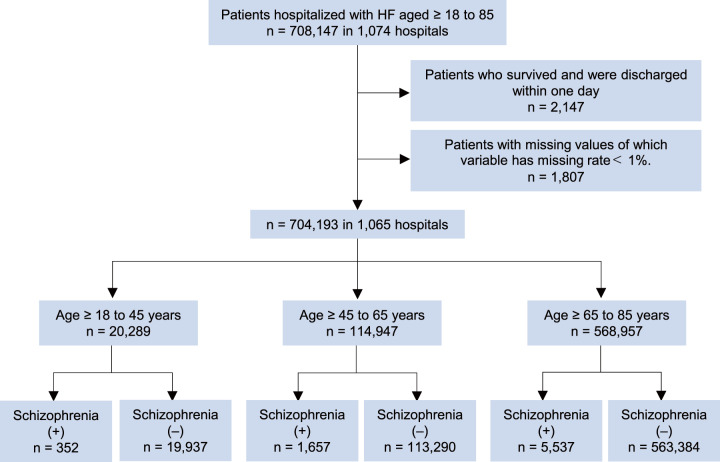
Flow diagram of the data filtering and stratification process. We excluded survived patients who survived and were discharged within 1 day. When the missing rate of a variable was less than 1%, we excluded the patients with that missing value. Ultimately, 704,193 patients across 1,065 hospitals were included for analysis. Following multiple imputation, the data were stratified into distinct age group.

A total of 704,193 patients hospitalized with HF were stratified by age: young age, ≥18 to 45 years (*n* = 20,289); middle age, ≥45 to 65 years (*n* = 114,947); and old age, ≥65 to 85 years (*n* = 568,957), and subsequently classified by the comorbidity of schizophrenia in each age group (Fig. 1). The prevalence of schizophrenia was 1.7 % in the young age, 1.4 % in the middle age and 0.97 % in the old age groups (Table 1). The patients with comorbid schizophrenia were more likely to be female, have a lower proportion of NYHA class I, II and III while having a higher proportion of NYHA class IV, require ventilator support, experience longer hospital stays, incur greater hospitalization costs and be admitted to non-training hospitals compared to those without comorbid schizophrenia across all age groups.

**Table 1.** Age-stratified baseline characteristics of HF patients with or without schizophrenia

	Age ≥18 to 45 years (*n* = 20,289)		Age ≥45 to 65 years (*n* = 114,947)		Age ≥65 to 85 years (*n* = 568,957)	
Characteristics	Schizophrenia (+) (1.7%, *n* = 352)	Schizophrenia (–) (98.2%, *n* = 19,937)	Schizophrenia (+) (1.4%, *n* = 1,657)	Schizophrenia (–) (98.5%, n = 113,290)	Schizophrenia (+) (0.97%, *n* = 5,573)	Schizophrenia (–) (99.0%, *n* = 563,384)
Patient characteristics						
Age (years)	38 (5.4)	37.6 (5.7)	56.1 (5.7)	57 (5.5)	76.4 (5.6)	76.6 (5.5)
Male (%)	61.0	71.8	64.6	75.3	52.4	59.6
BMI (kg/m^2^)	29.7 (9.5)	27.9 (8.2)	26.3 (6.6)	25.3 (5.8)	22.3 (4.4)	22.7 (4.7)
sBP < 100 mmHg (%)	16.8	17.9	14.9	14.6	12.9	13.3
Heart rate > 100 bpm (%)	60.1	53.4	49.1	44.0	38.6	32.9
NYHA I (%)	5.8	10.6	6.1	8.7	5.4	6.9
NYHA II (%)	21.2	28.5	23.8	27.1	22.6	26.7
NYHA III (%)	32.7	34.5	32.5	34.0	33.4	34.8
NYHA IV (%)	40.0	26.2	37.4	30.0	38.4	31.3
Charlson score	1 (1−2)	1 (1−3)	2 (1−3)	2 (1−3)	2 (1−3)	2 (1−3)
Diabetes (%)	22.1	25.3	32.1	38.6	22.4	32.8
Hypertension (%)	39.2	52.5	41.4	56.1	44.6	53.0
Dyslipidaemia (%)	11.9	18.3	14.6	26.3	14.8	23.3
Atrial fibrillation (%)	12.2	15.2	17.9	23.8	29.3	34.0
Smoking history (%)	53.6	52.4	57.4	62.6	43.6	45.4
Dialysis (%)	5.6	6.7	7.2	9.9	4.2	6.7
Ventilator (%)	27.8	19.0	25.9	23.4	23.0	22.8
IABP (%)	1.9	1.9	1.6	1.9	0.9	1.1
VA-ECMO (%)	0.5	0.8	0.3	0.4	0.1	0.2
Hospitalization costs (USD)	8130 (4333−14,954)	7590 (4575−12,468)	8296 (4911−14,079)	7654 (4803−12,808)	8232 (5468−12,925)	7567 (4927−12,256)
Duration of hospitalization (days)	17 (9−30)	16 (9−25)	17 (10−30)	15 (10−24)	20 (13−32)	17 (11−28)
Hospital characteristics						
Bed	515 (363−724)	527 (374−707)	489 (345−628)	472 (344−644)	430 (311−579)	424 (316−590)
Hospitalizations with HF per year	250 (178−345)	242 (163−356)	229 (159−322)	233 (159−332)	221 (150−311)	229 (155−326)
CV rehabilitation per year	4803 (2448−8201)	5099 (2644−8920)	4430 (2028−7476)	4763 (2455−8613)	4224 (2052−7476)	4616 (2321−8466)
Non-training facility (%)	8.6	7.8	11.6	9.7	14.4	11.8

Data are represented as mean (SD) if normally distributed, median (IQR) if non-normally distributed for numerical values, and % for categorical values. HF, heart failure; PCI, percutaneous coronary intervention; BMI, body mass index; sBP, systolic blood pressure; NYHA, New York Heart Association; IABP, intra-aortic balloon pumping; ECMO, extracorporeal membrane oxygenation; CV, cardiovascular; SD, standard difference; IQR, interquartile range.

### Schizophrenia as a risk factor for in-hospital mortality in non-elderly patients hospitalized with HF

**Figure 2.**
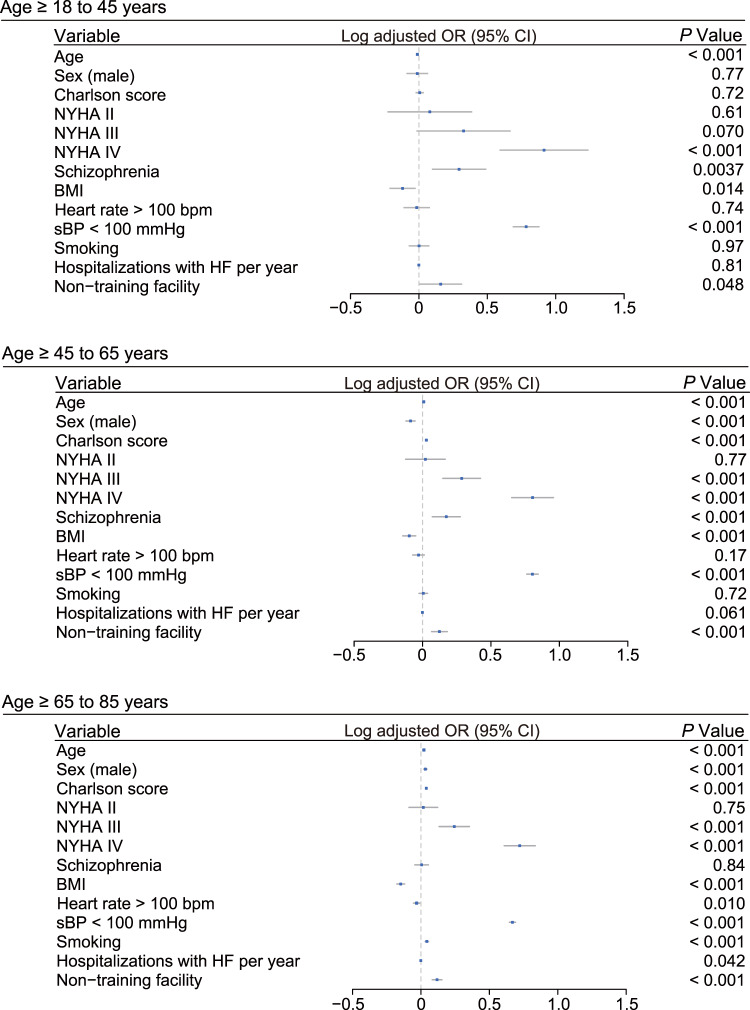
Effect of variables for in-hospital mortality. Adjusted OR for all in-hospital mortality was plotted with logarithmic scale. Error bar indicates 95% CI. BMI, body mass index; sBP, systolic blood pressure; NYHA, New York Heart Association; HF, heart failure; OR, odds ratio; CI, confidence interval.

We assessed the association between comorbid schizophrenia and in-hospital mortality in patients hospitalized with HF (Table 2). All in-hospital mortality of non-elderly HF patients with schizophrenia was significantly worse than those without schizophrenia: the mortality rate was 7.6% vs 3.5% and the adjusted odds ratio (OR) was 1.96 (95% confidence interval (CI): 1.24–3.10, *P* = 0.0037) in young adult patients aged ≥18 to 45 years; 6.2% vs 4.0% and 1.49 (95% CI: 1.17–1.88, *P* < 0.001) in middle-aged patients aged ≥45 to 65 years. We also evaluated 30-day in-hospital mortality, given its high mortality rate within the initial month following admission for patients with HF. The 30-day in-hospital mortality rate was 4.2% vs 2.7% and adjusted OR was 1.17 (95% CI: 0.64–2.12, *P* = 0.60) in young adult patients; 4.7% vs 3.0% and 1.40 (95% CI: 1.07–1.83, *P* = 0.012) in middle-aged patients. Conversely, all and 30-day in-hospital mortality in elderly patients with HF aged ≥ 65 to 85 years did not significantly differ between patients with and without schizophrenia: mortality rate was 8.9% vs 8.3% and adjusted OR was 1.01 (95% CI: 0.90–1.13, *P* = 0.84) for all in-hospital mortality; 6.0% vs 5.9% and 0.92 (95% CI: 0.81–1.05, *P* = 0.24) for 30-day in-hospital mortality. Effect of variables for all in-hospital mortality was depicted in Fig. 2. Collectively, schizophrenia was an independent risk factor for in-hospital mortality in non-elderly adult patients hospitalized with HF, but not in elderly patients.

**Table 2.** In-hospital mortality in HF patients with or without schizophrenia

	Age ≥18 to 45 years	Age ≥45 to 65 years	Age ≥65 to 85 years
	Schizophrenia (+) vs (–)	Schizophrenia (+) vs (–)	Schizophrenia (+) vs (–)
In-hospital mortality	OR (95% CI), *P* value	OR (95% CI), *P* value	OR (95% CI), *P* value
All	7.6 vs 3.5 % 1.96 (1.24−3.10), 0.0037	6.2 vs 4.0 % 1.49 (1.17−1.88), < 0.001	8.9 vs 8.3 % 1.01 (0.90−1.13), 0.84
30-day	4.2 vs 2.7% 1.17 (0.64−2.12), 0.60	4.7 vs 3.0 % 1.40 (1.07−1.83), 0.012	6.0 vs 5.9 % 0.92 (0.81−1.05), 0.24

First line in each row represents mortality rate. Adjusted ORs of all and 30-day in-hospital mortality were calculated for comorbidity of schizophrenia in age-stratified patients hospitalized with HF. OR, odds ratio; CI, confidence interval; HF, heart failure.

### Influence of comorbid schizophrenia on the prescription of cardioprotective medications in patients hospitalized with HF

To ascertain whether the quality of care is maintained in schizophrenia patients hospitalized with HF, the prescriptions of beta-blockers, angiotensin-converting enzyme (ACE) inhibitors or angiotensin II receptor blockers (ARBs), mineralocorticoid receptor antagonists (MRAs) and anticoagulants for atrial fibrillation (AF) were scrutinized as process-of-care measures (Table 3). The prescription of beta-blockers was significantly lower in patients with schizophrenia across all age groups: the prescription rate was 62.2% vs 73.1% and adjusted OR was 0.56 (95% CI: 0.44–0.71, *P* < 0.001) in young adult patients aged ≥18 to 45 years; 63.1% vs 74.4% and 0.58 (95% CI: 0.52–0.64, *P* < 0.001) in middle-aged patients aged ≥45 to 65 years; and 60.6% vs 63.2% and 0.87 (95% CI: 0.82–0.93, *P* < 0.001) in elderly patients aged ≥65 to 85 years. The prescription of ACE inhibitors or ARB was also significantly lower in patients with schizophrenia across all age groups: prescription rate was 59.6% vs 67.9% and adjusted OR was 0.68 (95% CI: 0.54–0.86, *P* = 0.0013) in young adult patients; 56.1% vs 65.2% and 0.67 (95% CI: 0.61–0.75, *P* < 0.001) in middle-aged patients; and 50.0% vs 55.9 % and 0.77 (95% CI: 0.73–0.82, *P* < 0.001) in elderly patients. No such influence of comorbid schizophrenia was observed for the prescription of MRAs and anticoagulant for AF. Consequently, prescription of beta-blockers and ACE inhibitors or ARBs was significantly lower in schizophrenia patients hospitalized with HF.

**Table 3.** Cardiovascular medications as process-of-care measures in HF patients with or without schizophrenia

	Age ≥18 to 45 years	Age ≥45 to 65 years	Age ≥65 to 85 years
	Schizophrenia (+) vs (–)	Schizophrenia (+) vs (–)	Schizophrenia (+) vs (–)
Medication	OR (95% CI), *P* value	OR (95% CI), *P* value	OR (95% CI), *P* value
Beta blockers	62.2 vs 73.1% 0.56 (0.44−0.71), < 0.001	63.1 vs 74.4% 0.58 (0.52−0.64), < 0.001	60.6 vs 63.2 % 0.87 (0.82−0.93), < 0.001
ACE inhibitors or ARBs	59.6 vs 67.9% 0.68 (0.54−0.86), 0.0013	56.1 vs 65.2% 0.67 (0.61−0.75), < 0.001	50.0 vs 55.9 % 0.77 (0.72−0.81), < 0.001
MRAs	59.9 vs 58.0% 1.04 (0.82−1.31), 0.71	55.9 vs 51.8% 1.07 (0.97−1.19), 0.15	50.9 vs 45.3% 1.20 (1.13−1.27), < 0.001
Anticoagulants for AF	91.0 vs 91.6% 0.88 (0.20−3.82), 0.87	89.5 vs 93.3% 0.59 (0.34−1.02), 0.067	86.6 vs 89.8% 0.71 (0.57−0.88), 0.0028

First line in each row represents prescription rate. Adjusted ORs of prescription of cardiovascular medications were calculated for comorbidity of schizophrenia in age-stratified patients hospitalized with HF. Anticoagulants were assessed for patients with AF. ACE, angiotensin-converting enzyme; ARBs, angiotensin II receptor blockers; MRAs, mineralocorticoid receptor antagonists; AF, atrial fibrillation; HF, heart failure.

## Discussion

In this study, we investigated in-hospital mortality and cardiovascular treatment during hospitalization for HF among age-stratified patients with schizophrenia. We identified schizophrenia as a risk factor for in-hospital mortality in non-elderly adult patients hospitalized with HF but not in elderly patients. Furthermore, prescriptions of beta-blockers and ACE inhibitors or ARBs were found to be lower in patients with schizophrenia. These findings suggest that comorbidity of schizophrenia exacerbates in-hospital prognosis and undermines the prescription of cardiovascular medications for patients hospitalized with HF.

Schizophrenia was identified as a significant risk factor for in-hospital mortality in non-elderly adult patients hospitalized with HF but not in elderly patients. In young adults, the prevalence of HF is inherently low due to its aetiology being predominantly reliant on age-related factors. Given the nature of HF, schizophrenia might exert a substantial impact on the pathology of HF and subsequent mortality in young adult patients. In contrast, the impact of schizophrenia might be attenuated in elderly patients with HF by a selection bias due to a shorter lifespan for schizophrenia patients than the general population. It is supported by the lower prevalence rate of schizophrenia in elderly group compared to younger groups.

The process of care is assessed using various metrics, including the prescription rates of beta-blockers, ACE inhibitors or ARBs, MRAs and anticoagulants for AF during hospitalization for HF (Albert *et al.*, 2009; Ellrodt *et al.*, 2013; Heidenreich *et al.*, 2020, 2009; Hernandez *et al.*, 2010). Patients with severe mental illness are less likely to receive guideline-recommended medications for CVD, consequently exacerbating mortality (Attar *et al.*, 2020; Kugathasan *et al.*, 2018; Mitchell *et al.*, 2012). Similarly, the quality of care for HF is suboptimal in patients with schizophrenia, and the prescription of beta-blockers for HF is lower in these patients (Jorgensen *et al.*, 2017). We observed significantly lower prescriptions of beta-blockers and ACE inhibitors or ARBs in patients with schizophrenia. Reduced prescription of these medications before admission may have influenced in-hospital mortality in patients hospitalized with HF.

There are several limitations inherent to the nature of the data. First, the data did not include laboratory data. Second, HF types were not classified by ejection fraction (EF). Consequently, the process of care measures was evaluated for patients with HF, including those with preserved EF, which lacks compelling cardioprotective evidence for the treatment of beta-blockers, ACE inhibitors or ARBs, and MRAs. Third, the data did not encompass the cause of death. Lastly, the severity of schizophrenia was not examined.

In conclusion, we demonstrated that comorbidity of schizophrenia is an independent risk factor for in-hospital mortality and reduces the prescription of cardioprotective medications in non-elderly patients hospitalized with HF. These findings underlie the necessity for tailored care and management of HF in schizophrenia patients during hospitalization.

## Data Availability

The authors are not permitted to share the data publicly. The data can be obtained by contacting The Japanese Circulation Society (the data provider) based on reasonable request (https://www.j-circ.or.jp/jittai_chosa/).
